# Supplementary material for: Promoting language and literacy through shared book reading in the NICU: A scoping review
Source: PLoS One. 2025 Mar 6;20(3):e0318690. doi: 10.1371/journal.pone.0318690 (PMC11884721; doi:10.1371/journal.pone.0318690)
Supplement: S1 Table — (DOCX) [file pone.0318690.s001.docx]

| Citation | Setting and Population | Study Design | Intervention | Results |
| --- | --- | --- | --- | --- |
| Canarte et al. (2022) | Caretakers of infants in the NICU. The pre- survey consisted of 50 families (2019). Post-survey data collection consisted of 12 families due to COVID-19. | Pre- and Post- Survey. | The R.E.A.D. (Read to, Enjoy, And Develop) Your Baby program. The campaign consists of giving baby books to families with an infant in the NICU >7 days and bi-weekly for chronically hospitalized infants. | Shared reading was occurring for only a minority of infants in the NICU before the R.E.A.D. Your Baby campaign. Significant changes were observed in survey responses centered around language use (talking, singing, or reading) by parents in the NICU from 25.2% in the pre-survey to 41.7% in the post-survey (p = .024). Shared reading in the NICU increased from 22% of parents in the pre-survey to 91.7% in the post-survey (p = .00000). |
| Fraser et al. (2023) | Parents of infants (n=35) in a surgical NICU. | Cross-  sectional non-identical sampling survey pre- and post-  intervention. | Read-a-thon program. | There was an increase in reading recommended post-  intervention (86%) compared to pre-  intervention (76%). Reading in the first week of life significantly increased from 43%  pre-intervention to 90% of post-  intervention  (p = 0.022). Encouraging factors for reading included privacy, having books available and being able to hold their baby. Discouraging factors included other people hearing them read, if their baby is sleeping, and if there were too many people near their baby. The discouraging factor of other people hearing them read decreased significantly from 35% pre-  intervention to 8% post-  intervention  (p = 0.042). |
| Jain et al. (2021) | 317 infants: unexposed comparison group (n = 187); intervention group (n = 130). | Parent survey. | Bookworms book-sharing reading intervention in the NICU. | Parents in the intervention group reported reading aloud > or equal to 3-4 days per week more to their infants in the NICU compared to parents in the unexposed comparison group (34.5% vs 51.5%; p = .002; aOR, 2.2; 95% CI, 1.2-4.0). |
| Kale & Deshpande (2023) | 18 infants, 7 or > days in NICU, Apgar score of more than 6 at first and fifth minute. | Pre- and Post- experimental study design. | Evaluated effectiveness of Reach Out and Read (ROR) on communication and generalized movement at a tertiary hospital in India over a period of 6 months. | Non-parametric statistics (Wilcoxon matched pair test) showed improvement in general movement over time (p = .0277; p = .0431). However, no significant increase in expressive or receptive communication outcomes (p>.05). |
| Lariviere & Rennick (2011) | 116 infants recruited in the NICU: Intervention group (n = 59); historical control group (n = 57). | Mixed methods design using a non-randomized, participant blinded intervention study using a historical control group. Pre- and Post- study design with survey and qualitative interviews. | Parental reading intervention in the NICU. Parents selected a book and read every day as they held their infant, at bedside, or in the incubator. | Increased frequent reading (majority reported reading more than three times per week) in the intervention group 55.9% compared to the control group 17.5% (p<.001); evident three months after discharge.  Parents reported a higher sense of control. |
| Latif et al. (2023) | 1,255 infants admitted to a level IV NICU and step-down NICU. | Qualitative (interviews, open-ended surveys, document reviews). | Read-a-Thon (Read-a-Latte) spanning 10 days. Books were distributed to families of all infants in the NICU. Healthcare professionals and caregivers were encouraged to read to infants. | A total of 663 reading sessions were logged during the 10-day Read-a-Thon. Six qualitative themes ​​were identified: Motivation, emotional response to the program, benefits and outcomes, barriers, facilitators, and future of literacy promotion in the NICU. |
| Levesque et al. (2018) | 98 preterm infants born between March 1 and December 31, 2015, < 37- week gestation. | Descriptive statistics (percent enrolled, read to, etc.) Anonymous parent survey. | Pilot study of Reach Out and Read (ROR). Parental reading with 95% of infants read to using books in the mothers’ primary language  (English, Spanish, Portuguese, French, Haitian Creole, Vietnamese). | The median percentage of families reading to their baby increased from 0% before and 59% after the ROR program. |
| Scala et al. (2018) | 18 Preterm infants (23–31-week gestation). | Prospective unblinded pre- and post- study design. | Parental (mothers and fathers) reading intervention in the NICU.  Comparison between live and recorded reading. | Fewer desaturation events (reduced Oxygen) of less than 85% during parental reading than prior to reading exposure (p=.0001). These effects persisted up to 1 hour after reading exposure. |
