# Supplementary material for: Promoting language and literacy through shared book reading in the NICU: A scoping review
Source: PLoS One. 2025 Mar 6;20(3):e0318690. doi: 10.1371/journal.pone.0318690 (PMC11884721; doi:10.1371/journal.pone.0318690)
Supplement: S1 Supporting Information — (DOCX) [file pone.0318690.s002.docx]

Supporting Information

**Database Search Strategies**

Searched December 8, 2023, Firefox browser

| **Databases** | **Records Retrieved** |
| --- | --- |
| **APA PsycINFO (EBSCOhost)** mapping turned off  (DE "Neonatal Intensive Care" OR "Neonatal intensive care unit*" OR "neonatal ICU*" OR NICU* OR Premature OR prematurity OR Preterm OR newborn* OR DE "Premature Birth") **AND** ("shared reading" OR "shared book reading" OR "Reach Out and Read" OR "reading aloud" OR "read aloud" OR "reading session*" OR "parental reading" OR "language exposure" OR "speech exposure")  Limits: Publication Date: 20030101-20231231, English language | 20 |
| **Child Development & Adolescent Studies (EBSCOhost)** mapping turned off  ("Neonatal intensive care unit*" OR "neonatal ICU*" OR NICU* OR Premature OR prematurity OR Preterm OR newborn*) **AND** ("shared reading" OR "shared book reading" OR "Reach Out and Read" OR "reading aloud" OR "read aloud" OR "reading session*" OR "parental reading" OR "language exposure" OR "speech exposure")  Limits: Publication Date: 20030101-20231231 | 2 |
| **CINAHL (EBSCOhost)** mapping turned off  (MH "Intensive Care Units, Neonatal" OR "Neonatal intensive care unit*" OR "neonatal ICU*" OR NICU* OR Premature OR prematurity OR Preterm OR newborn*) **AND** ("shared reading" OR "shared book reading" OR "Reach Out and Read" OR "reading aloud" OR "read aloud" OR "reading session*" OR "parental reading" OR "language exposure" OR "speech exposure")  Limits: Publication Date: 20030101-20231231, English language | 45 |
| **Embase.com** mapping turned off  ('neonatal intensive care unit'/de OR "Neonatal intensive care unit*" OR "neonatal ICU*" OR NICU* OR Premature OR prematurity OR 'prematurity'/exp OR Preterm OR newborn*) **AND** ("shared reading" OR "shared book reading" OR "Reach Out and Read" OR "reading aloud" OR "read aloud" OR "reading session*" OR "parental reading" OR "language exposure" OR "speech exposure") **AND** [english]/lim AND [2003-2023]/py | 78 |
| **ERIC (EBSCOhost)** mapping turned off  ("Neonatal intensive care unit*" OR "neonatal ICU*" OR NICU* OR Premature OR prematurity OR Preterm OR newborn*) **AND** ("shared reading" OR "shared book reading" OR "Reach Out and Read" OR "reading aloud" OR "read aloud" OR "reading session*" OR "parental reading" OR "language exposure" OR "speech exposure")  Limits: Publication Date: 20030101-20231231, English language | 6 |
| **Health Source: Nursing/Academic (EBSCOhost)** mapping turned off (DE "NEONATAL intensive care units" OR DE "PREMATURE infants" OR "Neonatal intensive care unit*" OR "neonatal ICU*" OR NICU* OR Premature OR prematurity OR Preterm OR newborn*) **AND** ("shared reading" OR "shared book reading" OR "Reach Out and Read" OR "reading aloud" OR "read aloud" OR "reading session*" OR "parental reading" OR "language exposure" OR "speech exposure")  Limits: Publication Date: 20030101-20231231 | 5 |
| **Linguistics and Language Behavior Abstracts (ProQuest)** abstract(("Neonatal intensive care unit*" OR ("neonatal icu*") ))  Limits: English | 24 |
| **PubMed** ("Neonatal intensive care unit*"[tw] OR "neo-natal intensive care unit"[tw] OR "neonatal ICU*"[tw] OR NICU*[tw] OR "Intensive Care Units, Neonatal"[Mesh] OR Premature[tw] OR prematurity[tw] OR Preterm[tw] OR newborn*[tw] OR "Infant, Newborn"[Mesh]) **AND** ("shared reading"[tw] OR "shared book reading"[tw] OR "Reach Out and Read"[tw] OR "reading aloud"[tw] OR "read aloud"[tw] OR "reading session*"[tw] OR "parental reading"[tw] OR "language exposure"[tw] OR "speech exposure"[tw] OR ("Reading"[Mesh] AND "Parent-Child Relations"[Mesh])) **AND** ("2003/01/01"[PDAT] : "2023/12/31"[PDAT]) **AND** english[Filter] | 81 |
| **Scopus (Elsevier)** TITLE-ABS-KEY("Neonatal intensive care unit*" OR "neonatal ICU*" OR NICU* OR Premature OR prematurity OR Preterm OR newborn*) **AND** TITLE-ABS-KEY("shared reading" OR "shared book reading" OR "Reach Out and Read" OR "reading aloud" OR "read aloud" OR "reading session*" OR "parental reading" OR "language exposure" OR "speech exposure") **AND** 2003-2023 **AND** english | 77 |
| Total number of citations uploaded to Covidence | 338 |
