# Supplementary material for: Promoting language and literacy through shared book reading in the NICU: A scoping review
Source: PLoS One. 2025 Mar 6;20(3):e0318690. doi: 10.1371/journal.pone.0318690 (PMC11884721; doi:10.1371/journal.pone.0318690)
Supplement: Attachment — Submitted filename: Response to Reviewers.docx [file pone.0318690.s004.docx]

November 22, 2024

Sanjoy Kumer Dey, M.D

Academic Editor

PLOS ONE

Dear Dr. Dey:

Thank you for the review of our manuscript, “Promoting Language and Literacy Through Shared Book Reading in the NICU: A Scoping Review,” [PONE-D-23-44086]. We are pleased to learn your decision of “minor revision”, inviting us to re-submit our manuscript for publication.

We address your points and those of the reviewers below.

**Journal Requirements**

1.	Please ensure that your manuscript meets PLOS ONE's style requirements, including those for file naming. The PLOS ONE style templates can be found at

https://journals.plos.org/plosone/s/file?id=wjVg/PLOSOne_formatting_sample_main_body.pdf and

<https://journals.plos.org/plosone/s/file?id=ba62/PLOSOne_formatting_sample_title_authors_affiliations.pdf>

We have revised the manuscript to meet PLOS ONE’s style requirements.

2.	Thank you for stating the following financial disclosure: "This research has been funded by a grant from the Deal Center for Early Language and Literacy to Lama K. Farran." Please state what role the funders took in the study. If the funders had no role, please state: ""The funders had no role in study design, data collection and analysis, decision to publish, or preparation of the manuscript."" If this statement is not correct you must amend it as needed. Please include this amended Role of Funder statement in your cover letter; we will change the online submission form on your behalf.

We have stated the following financial disclosure: “This research has been funded by a grant from the Deal Center for Early Language and Literacy to Lama K. Farran.”

We have stated the following role of the funders in the study: “The funders had no role in study design, data collection and analysis, decision to publish, or preparation of the manuscript.”

3. We note that your Data Availability Statement is currently as follows: All relevant data are within the manuscript and its Supporting Information files. Please confirm at this time whether or not your submission contains all raw data required to replicate the results of your study. Authors must share the “minimal data set” for their submission. PLOS defines the minimal data set to consist of the data required to replicate all study findings reported in the article, as well as related metadata and methods (<https://journals.plos.org/plosone/s/data-availability#loc-minimal-data-set-definition>).

We included our search strategy, including inclusionary and exclusionary criteria, as well as provided our PRISMA checklist. Additionally, we presented our findings, listing the number of articles meeting criteria for inclusion. Given the nature of this manuscript being a scoping review, the replicability of the findings is contingent upon using the same methodology at this date and time.

4.	Please ensure that you include a title page within your main document. You should list all authors and all affiliations as per our author instructions and clearly indicate the corresponding author.

The title page is included listing all authors and their respective affiliations. We have specified the corresponding author.

5.	Please include your tables as part of your main manuscript and remove the individual files. Please note that supplementary tables (should remain/ be uploaded) as separate ""supporting information"" files

We included the table as part of the main manuscript.

6.	Please include captions for your Supporting Information files at the end of your manuscript, and update any in-text citations to match accordingly. Please see our Supporting Information guidelines for more information: <http://journals.plos.org/plosone/s/supporting-information>.

We included captions for our supporting information files at the end of our manuscript and updated the corresponding in-text citations.

7.	Please review your reference list to ensure that it is complete and correct. If you have cited papers that have been retracted, please include the rationale for doing so in the manuscript text, or remove these references and replace them with relevant current references. Any changes to the reference list should be mentioned in the rebuttal letter that accompanies your revised manuscript. If you need to cite a retracted article, indicate the article’s retracted status in the References list and also include a citation and full reference for the retraction notice.

We reviewed the reference list and none of the publications have been retracted. We have added new references as suggested by the reviewers.

8.	While revising your submission, please upload your figure files to the Preflight Analysis and Conversion Engine (PACE) digital diagnostic tool, https://pacev2.apexcovantage.com/. PACE helps ensure that figures meet PLOS requirements. To use PACE, you must first register as a user. Registration is free. Then, login and navigate to the UPLOAD tab, where you will find detailed instructions on how to use the tool. If you encounter any issues or have any questions when using PACE, please email PLOS at figures@plos.org. Please note that Supporting Information files do not need this step.

We used PACE and verified that our figures meet PLOSONE requirements.

**Response to Reviewer #1’s comments**

1.	The abstract is written in an unstructured format. If the journal guidelines permit, please revise it to a structured format with the following headings: Objectives, Methods, Results, Conclusion, Implications for practice, and Keywords.

We revised the abstract to include the suggested headings.

2.	Conclusions: Consider adding a sentence about the practical implications of your findings for healthcare providers.

We added a sentence on the implications of our findings and the importance of including both caregivers and healthcare providers.

3.	Introduction section: The introduction section is very lengthy. I strongly recommend it be written more concisely. Additionally, the introduction should address the necessity of supporting parents by healthcare providers, including nurses. For example, the authors can refer to the following articles on this topic:

https://journals.sagepub.com/doi/full/10.1177/17455057221104674

https://onlinelibrary.wiley.com/doi/full/10.1155/2021/6697659

We have shortened the introduction and addressed the necessity of healthcare providers’ (including nurses’) support for parents in the NICU. We cited the two articles the reviewers suggested.

4.	The discussion section provides a comprehensive overview of the study's aim, findings, and implications. However, some areas could benefit from further clarification, structural improvements, and more detailed integration of the study results with the existing literature.

We have revised the discussion section to reflect a deeper integration of the study results with the existing literature.

**Response to Reviewer #2’s comments**

1.	The manuscript is well written and presented and could be accepted for publication in its current form.

We appreciate your comments and positive feedback.

Sincerely,

Lama K. Farran

Susan Brasher

Sharon Leslie
